# Inflammatory Markers in Non-Obese Women with Polycystic Ovary Syndrome Are Not Elevated and Show No Correlation with Vitamin D Metabolites

**DOI:** 10.3390/nu14173540

**Published:** 2022-08-27

**Authors:** Abu Saleh Md Moin, Thozhukat Sathyapalan, Stephen L. Atkin, Alexandra E. Butler

**Affiliations:** 1 Royal College of Surgeons in Ireland Bahrain, Adliya 15503, Bahrain; 2 Academic Endocrinology, Diabetes and Metabolism, Hull York Medical School, Hull YO10 5DD, UK

**Keywords:** polycystic ovary syndrome, inflammation, vitamin D_3_, matrix metalloproteinases

## Abstract

Introduction. Chronic low-grade inflammation is a characteristic of women with polycystic ovary syndrome (PCOS), although this may be obesity-driven rather than an intrinsic facet of PCOS; furthermore, vitamin D deficiency, another common feature of PCOS, is reported to have an association with increased inflammation. Therefore, circulating inflammatory protein levels and circulating levels of vitamin D may be linked in PCOS, though it is unclear which vitamin D metabolites may be important. Methods. We measured plasma levels of 24 inflammatory proteins and 12 matrix metalloproteinases (proteins modulated by the inflammatory process) by slow off-rate modified aptamer (SOMA)-scan plasma protein measurement in weight and aged-matched non-obese non-insulin resistant PCOS (n = 24) and control (n = 24) women. Inflammatory proteins and matrix metalloproteinases were correlated to 25-hydroxy vitamin D_3_ (25(OH)D_3_), its epimer 25-hydroxy-3epi-vitamin D (3epi25(OH)D) and the active 1,25-dihydroxyvitamin D_3_ (1,25(OH)_2_D_3_) as measured by gold standard isotope-dilution liquid chromatography tandem mass spectrometry. Results. PCOS women had both an elevated free androgen index and circulating anti-mullerian hormone, though insulin resistance was comparable to controls. C-reactive protein, as a standard circulatory marker of inflammation, was comparable between cohorts. Levels of circulating inflammatory proteins and matrix metalloproteinases were not different between the PCOS and control women, with no correlation of 25(OH)D_3,_ 1,25(OH)_2_D_3_ or 3epi25(OH)D with any of the inflammatory proteins. Conclusion. In a non-obese PCOS population matched for age and insulin resistance, circulating inflammatory proteins and matrix metalloproteinases were not elevated and did not correlate with 25(OH)D_3,_ its epimer 3epi25(OH)D or 1,25(OH)_2_D_3_ in either control or PCOS women, indicating that the inflammatory response is absent and the vitamin D-metabolite independent in non-obese women with PCOS.

## 1. Introduction

In women with polycystic ovary syndrome (PCOS), the prevalence of type 2 diabetes, hypertension and cardiovascular disease is enhanced [1] and, whilst the mechanisms are still unclear, inflammation and obesity have been implicated [2]. Increased inflammation, as most commonly adjudged by elevations in C-reactive protein (CRP) [3], is associated with PCOS [4,5] and has been reported even in normal weight women with PCOS (though at admittedly lower levels that their obese counterparts) [6], although insulin resistance was not taken into account. Contradictory studies, however, have reported no difference in circulatory CRP between women with or without PCOS [7,8]. A recent systematic review with meta-analysis, aimed at determining whether the inflammation in PCOS women was intrinsic to the disease or conversely related to the associated adiposity, concluded that circulatory CRP is elevated in PCOS women and that this was independent of obesity [9]. Furthermore, raised circulating levels of tumor necrosis factor alpha (TNFα) have been reported to be present in non-obese women with PCOS [10].

Matrix metalloproteinases (MMPs) are protein markers of M2 macrophages that are considered to be important in PCOS pathogenesis [11]. MMPs, as well as tissue inhibitors of matrix metalloproteinases (TIMPs), are reported to be involved in ovarian physiology (follicular rupture and oocyte release) and pathophysiology [12]. For instance, an imbalance of MMPs and TIMPs, including reduced concentrations of TIMP-2 and elevated MMP-2/TIMP-2 and MMP-9/TIMP-1 ratios have been observed in PCOS [13]. Whilst there have been conflicting reports as to whether MMP2 and/or MMP9, for example, are elevated [14,15,16], unchanged [13], or reduced [17] in PCOS, they have been shown to correlate with BMI this significance was lost when BMI was accounted for [18]. MMPs, together with TIMPs, are key components of the immune response, regulating signalling downstream of the tumor necrosis factor (TNF) and interleukin 6 (IL6) receptors, key elements in inflammatory processes [19].

Clinically, this inflammatory process in PCOS may be of major importance as it is associated and connected with obesity comorbidities, such as endothelial dysfunction, atherosclerosis, and coronary heart disease [20]. In addition, it has been suggested that a uterine hyperinflammatory state is present in PCOS that may be responsible for pregnancy complications such as miscarriage and placental insufficiency [20].

Despite the prevalence of disease, the mechanisms underlying PCOS development are unclear. Vitamin D deficiency/insufficiency is extremely common in women with PCOS, 67–85% being severely deficient, and low levels of vitamin D have been correlated with obesity, insulin resistance and testosterone levels [21,22]. Vitamin D insufficiency/deficiency has been strongly linked to a variety of negative health outcomes, examples being osteoporosis and an increased incidence of type 2 diabetes with poorer glycemic control, as well as cancer, cardiovascular disease, autoimmune diseases and depression; in addition, an increase in mortality has been documented that can be circumvented with the use of vitamin D supplements [23,24]. However, some research has suggested that vitamin D deficiency does not exacerbate these features of cardiovascular risk in PCOS [25]. Studies indicate that vitamin D supplementation (commonly given in the form of oral vitamin D_2_ supplements) are beneficial in countering insulin resistance and steroidogenesis of estradiol and progesterone in obese PCOS women [26,27]. A systematic review with meta-analysis suggested that vitamin D deficiency was associated with insulin resistance, but the significance was lost when BMI was accounted for [28]. While inflammation is accepted as an underlying factor in a multitude of chronic diseases, how much of an influence vitamin D has on inflammation is still poorly defined and indeed vitamin D deficiency/insufficiency may, in fact, be a consequence of chronic inflammation instead of the converse [29].

**Figure 1:**
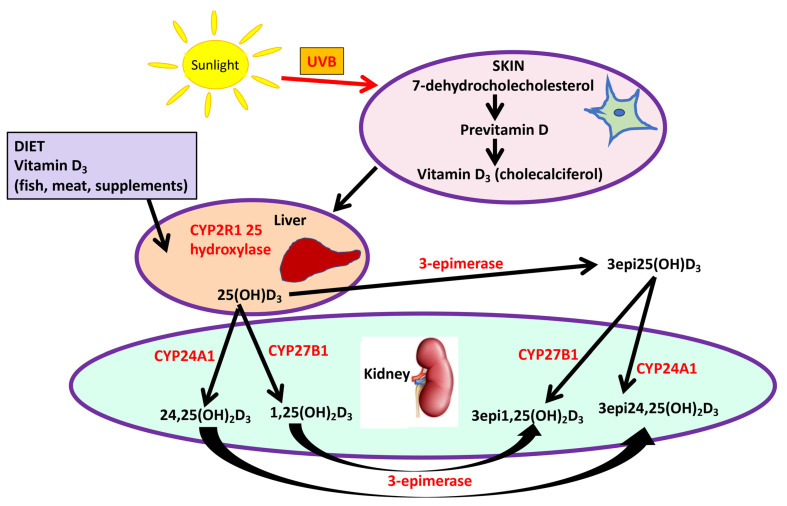
A schematic illustrating the sources (diet and sunlight) and metabolism in skin, liver and kidney of vitamin D_3_ metabolites.

As well as through dietary intake in such foods as eggs and dairy products, vitamin D_3_ (cholecalciferol) is endogenously produced and subsequently hydroxylated to vitamin D_3_ (25(OH)D_3_) by multiple hepatic 25-hydroxylases [30,31]. Next, 25(OH)D_3_ is transported to the kidney where it is either converted to the active 1,25(OH)_2_D by 1 alpha hydroxylase, or to the inactive 24,25(OH)_2_D [32] (Figure 1); however, 1,25(OH)_2_D is additionally produced in extrarenal tissues where it may act locally. Moreover, 3-epimerase converts 25(OH)D_3_ to 3epi25(OH)D_3_ [30,33] that may be measured by less specific methods than tandem mass spectroscopy for the measurement of 25(OH)D_3_, leading to its overestimation [34,35]. Little is known about vitamin D epimers, epimers being defined as having identical molecular weight but stereochemical differences relative to the parent molecule. There are few studies on 3epi25(OH)D [35,36] and generally it appears less active than 25(OH)D_3_; however, the 3-epimer may suppress parathyroid hormone (PTH) as actively as does 1,25(OH)_2_D_3_ [30,37].

The confounding effects of associated obesity in PCOS may have obscured whether the underlying pathophysiology of PCOS has independent effects on chronic inflammation and whether this is exacerbated by vitamin D deficiency. It is well recognised that obesity can worsen vitamin D deficiency by decreasing its bioavailability, as vitamin D is sequestered in adipose tissue [38]. Therefore, to circumvent the confounding effect of obesity, this study was undertaken in a non-obese PCOS population versus controls matched for BMI and insulin resistance. We sought to determine, firstly, whether the levels of circulatory inflammatory proteins differed between these non-obese PCOS and control women and, secondly, whether the levels of circulatory inflammatory proteins correlated with, and therefore may be modulated by, vitamin D metabolites.

## 2. Materials and Methods

This study was approved by The Yorkshire and The Humber NRES ethical committee, UK in February 2003 (approval number 02/03/043).

We determined plasma levels of 24 inflammatory proteins and 12 matrix metalloproteinases in PCOS (n = 24) and control (n = 24) women attending the Hull IVF clinic [39]. PCOS and control women were matched for age and body mass index (BMI). Table 1 depicts relevant demographic data for both the PCOS and control cohorts [39]. The Rotterdam consensus was used for the diagnosis of PCOS; “these criteria are (1) clinical and biochemical hyperandrogenaemia, requiring a Ferriman-Gallwey score of >8 and a free androgen index of >4, respectively, (2) oligomenorrhea or amenorrhea and (3) polycystic ovaries seen on transvaginal ultrasound” [40]. None of the study participants had any other illness or condition and all participants were required to be medication-free for nine months preceding study enrolment. All participants underwent testing to rule out the presence of the following conditions: androgen-secreting tumour, Cushing’s disease, non-classical 21-hydroxylase deficiency or hyperprolactinemia.

In order to account for seasonal fluctuations in levels of vitamin D metabolites, sampling for vitamin D was performed during the period of March to September, thereby allowing vitamin D targets to be achieved through just 9 min of sunlight exposure daily in Northern England [41]. None of the women included in the study were consuming vitamin D supplements, and these women had not taken any supplements in the 6-month period prior to study enrolment (as this was one of the exclusion criteria).

Fasting bloods were centrifuged at 3500× *g* for 15 min, aliquoted and frozen at −80 °C prior to analysis. The blood samples were analysed for plasma glucose using a Synchron LX20 analyser (Beckman–Coulter, High Wycombe, UK), sex hormone binding globulin (SHBG) and insulin using a DPC Immulite 200 analyser (Euro/DPC, Llanberis, UK). To calculate the free androgen index (FAI), total testosterone was divided by SHBG, and then multiplied by 100. The homeostasis model assessment (HOMA-IR) was used to determine insulin resistance (IR).

Levels of serum vitamin D levels were determined using gold standard isotope-dilution liquid chromatography tandem mass spectrometry (LC-MS/MS) as detailed previously [36]. “Briefly, internal standards (d6-1calcitriol (1.5 ng/mL), d6-25OHD_3_ (50 ng/mL) and d6-25OHD_2_ (20 ng/mL) were added into calibration standards, quality control or serum samples and kept for 30-min to reach equilibrium. Pre-treated samples were loaded onto ISOLUTE^®^ supported liquid extraction (SLE+) columns (Biotage, Uppsala, Sweden), followed by elution with n-heptane into a collection tube containing 0.25 mg/mL PTAD solution in ethyl acetate and heptane (8:92 *v*/*v*). The derivatised extracts were measured by LC-MS/MS” [39].

Circulating levels of inflammatory proteins and matrix metalloproteinases were measured by slow off-rate modified aptamer (SOMA)-scan plasma protein analysis (Somalogic, Boulder, CO, USA); this methodology has been detailed previously [42]. In line with the manufacturer’s recommendation, standard samples were included in each plate to allow for appropriate calibration and normalisation of raw fluorescent intensities, as has been delineated previously [43].

We measured levels of the following 24 plasma inflammatory proteins: Azurocidin (AZU1), C-C motif chemokine 19 (CCL19), CD40 ligand (CD40LG), C-type lectin domain family 7 member A (CLEC7A), C-X-C motif chemokine 10 (CXCL10), Fibroblast growth factor 8 (FGF8), High mobility group protein B1 (HMGB1), Interferon gamma (IFN-g), Interleukin (IL)1alpha, Interleukin-1 beta (IL1B), IL5, IL6, IL10, IL12, IL17, IL34, Interleukin 10 receptor beta subunit (IL10RB), Prostaglandin G/H synthase 2 (PTGS2), Protein kinase C zeta type (PRKCZ), Protein S100-A9 (S100A9), Ribosomal protein S6 kinase alpha-5 (RPS6KA5), Serine/threonine-protein kinase (TBK1), Sialoadhesin (SIGLEC1) and tumour necrosis factor alpha (TNF-a). In addition, we measured all 12 members of the matrix metalloproteinase family that are included in Somascan panel: Matrix metalloproteinases (MMPs) MMP1, MMP2, MMP3, MMP7, MMP8, MMP9, MMP10, MMP12, MMP13, MMP14, MMP16 and MMP17 (Table 2). Thus, a total of 36 inflammation-related proteins are presented here.

## 3. Statistics

A power analysis (nQuery version 9, Dotmatics, Statsol, San Diego, CA, USA) was undertaken for TNF alpha protein that had been previously reported in PCOS [10]. For 80% power and a significance level (alpha) of 0.05 with a common standard deviation of 0.26, the number of subjects required was 12; however, as the power could not be calculated for the other proteins, 48 subjects were recruited. Trends in the data were assessed for normality both visually and statistically. Student’s t-tests were used wherever the data was normally distributed; where data that was not normally distributed according to the Kolmogorov–Smirnov Test, a Mann–Whitney (non-parametric test) was utilised. Inflammatory protein and vitamin D metabolite levels were correlated; all statistics were undertaken with Graphpad Prism (Dotmatics, San Diego, CA, USA). A *p*-value of <0.05 was considered significant.

## 4. Results

Table 1 depicts pertinent demographic and biochemical data for the age and BMI-matched non-obese PCOS and control women (n = 24). Insulin resistance and C-reactive protein (CRP) were not different between cohorts. PCOS subjects had a raised free androgen index and raised anti-mullerian hormone, in accord with the diagnosis of PCOS. Levels of vitamin D metabolites were not different between cohorts for 25(OH)D_3,_ its epimer 3epi25(OH)D or 1,25(OH)_2_D_3._ Levels of vitamin D metabolites did not correlate with BMI or age for either group or for the total group. No significant correlation existed between any of the vitamin D metabolites and estimated glomerular filtration rate (eGFR).

The results of the inflammatory proteins are shown in Table 2 for both the non-obese PCOS and control subjects. In these non-obese, non-insulin resistant PCOS women, no difference was found between PCOS and controls for the levels of the following 24 plasma inflammatory proteins: CXCL10, IL5, AZU1, CLEC7A, TBK1, PRKCZ, RPS6KA5, CD40LG, IL34, HMGB1, S100A9, IL1B, CCL19, SIGLEC1, IL10RB, PTGS2, FGF8, IL6, IL10, IL12, IL17, IFN-g, TNF-a.

In these non-obese, non-insulin resistant PCOS women, no difference was found between PCOS and controls for the levels of the following 12 members of the matrix metalloproteinase family: MMP1, MMP2, MMP3, MMP7, MMP8, MMP9, MMP10, MMP12, MMP13, MMP14, MMP16 and MMP17 (Table 2). Thus, this study revealed no difference in the levels of the 36 inflammation-related proteins presented here between controls and non-obese PCOS subjects.

**Figure 2:**
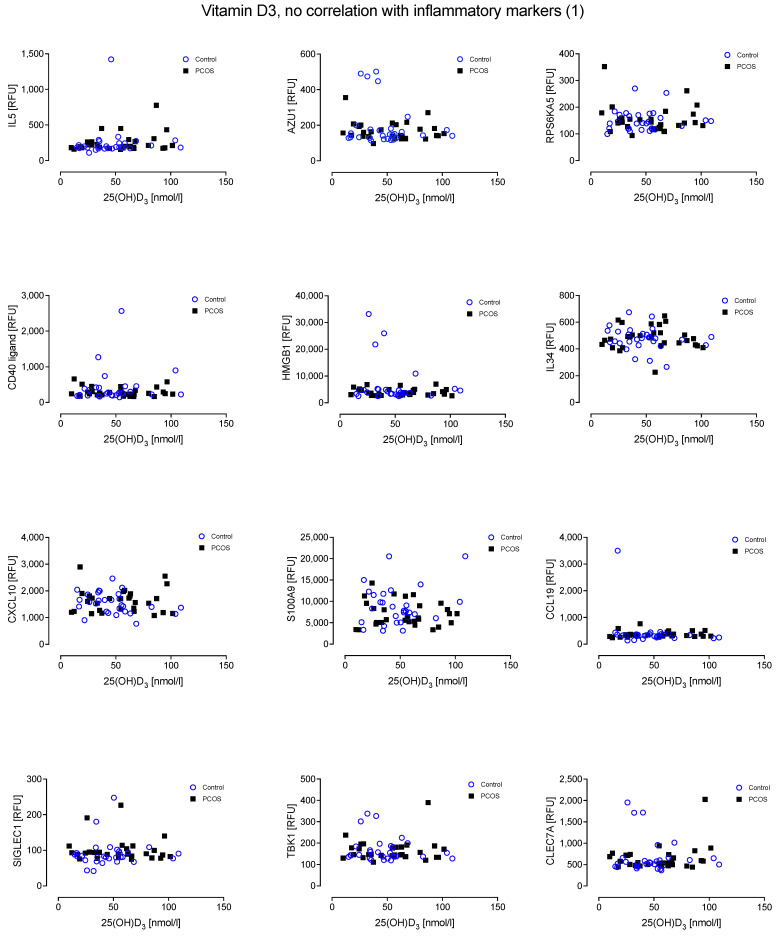
No relationship was found between Vitamin D_3_ and the inflammatory markers Interleukin-5 (IL5), Azurocidin (AZU1), Ribosomal protein S6 kinase alpha-5 (RPS6KA5), CD40 ligand (CD40LG), High mobility group protein B1 (HMGB1), Interleukin-34 (IL34), C-X-C motif chemokine 10 (CXCL10), Protein S100-A9 (S100A9), C-C motif chemokine 19 (CCL19), Sialoadhesin (SIGLEC1), Serine/threonine-protein kinase (TBK1) and C-type lectin domain family 7 member A (CLEC7A).

**Figure 3:**
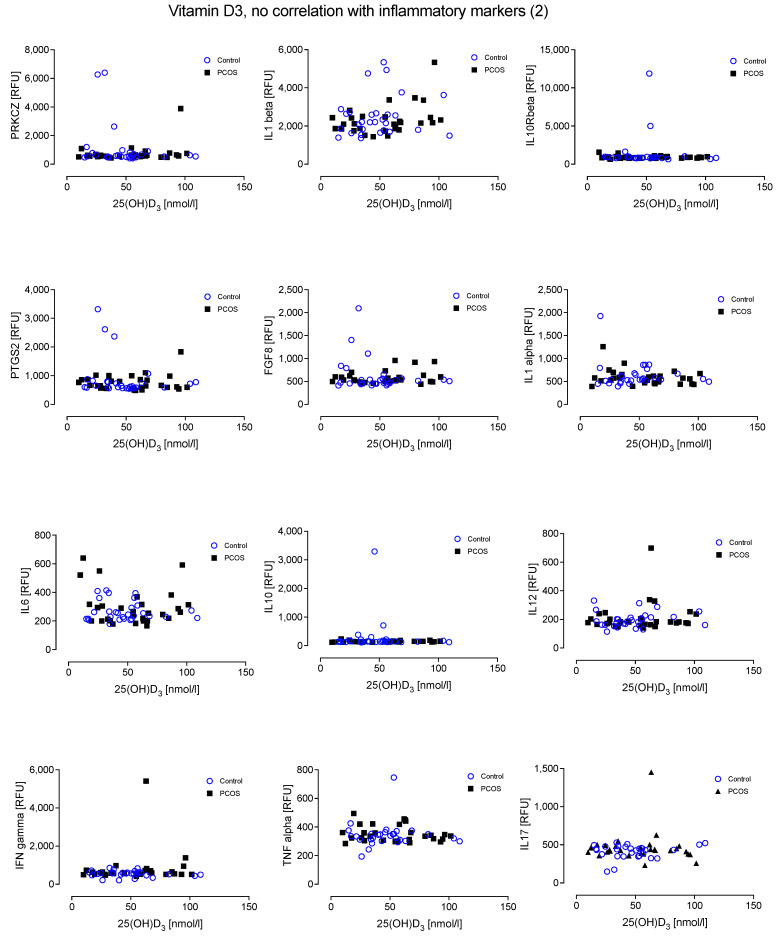
No relationship was found between Vitamin D_3_ and the inflammatory markers Protein kinase C zeta type (PRKCZ), Interleukin-1 beta (IL1beta), Interleukin 10 receptor beta subunit (IL10Rbeta), Prostaglandin G/H synthase 2 (PTGS2), Fibroblast growth factor 8 (FGF8), Interleukin-1 alpha (IL1alpha), IL6, IL10, IL12, Interferon gamma (IFN-g), Tumour necrosis factor alpha (TNF-a) and IL17.

Furthermore, there was no relationship evident between the inflammatory proteins and levels of 25(OH)D_3_ in either cohort (Figure 2 and Figure 3).

**Figure 4:**
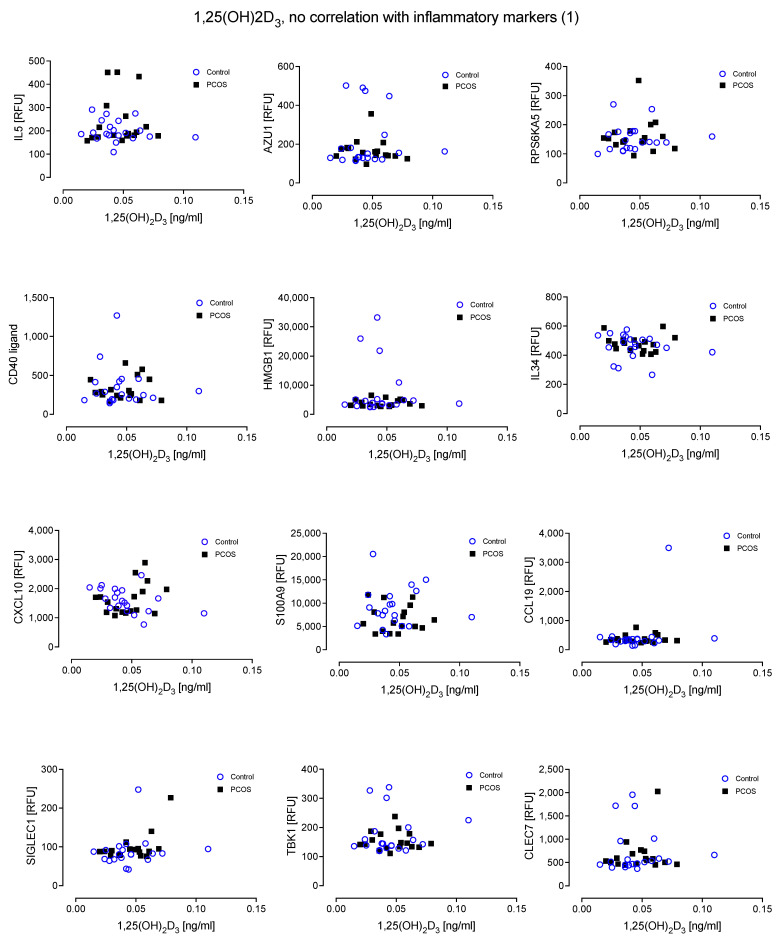
No relationship was found between 1,25(OH)_2_D_3_ and the inflammatory markers Interleukin-5 (IL5), Azurocidin (AZU1), Ribosomal protein S6 kinase alpha-5 (RPS6KA5), CD40 ligand (CD40LG), High mobility group protein B1 (HMGB1), Interleukin-34 (IL34), C-X-C motif chemokine 10 (CXCL10), Protein S100-A9 (S100A9), C-C motif chemokine 19 (CCL19), Sialoadhesin (SIGLEC1), Serine/threonine-protein kinase (TBK1) and C-type lectin domain family 7 member A (CLEC7A).

**Figure 5:**
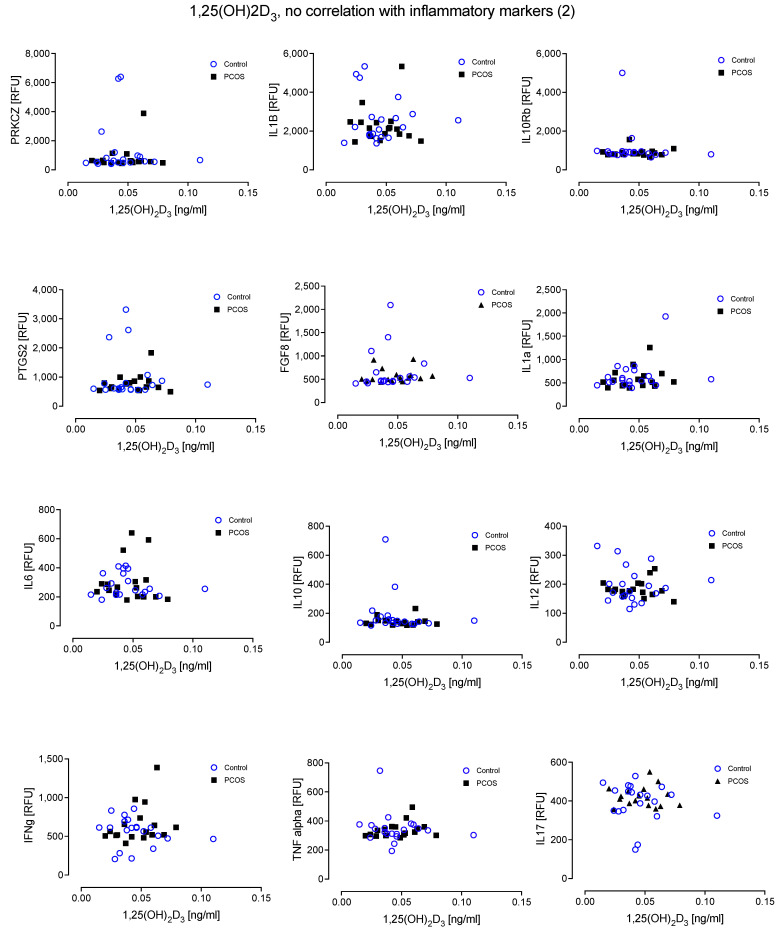
No relationship was found between 1,25(OH)_2_D_3_ and the inflammatory markers Protein kinase C zeta type (PRKCZ), Interleukin-1 beta (IL1beta), Interleukin 10 receptor beta subunit (IL10Rbeta), Prostaglandin G/H synthase 2 (PTGS2), Fibroblast growth factor 8 (FGF8), Interleukin-1 alpha (IL1alpha), IL6, IL10, IL12, Interferon gamma (IFN-g), Tumour necrosis factor alpha (TNF-a) and IL17.

There was no relationship evident between the inflammatory proteins and levels of active 1,25(OH)_2_D_3_ in either cohort (Figure 4 and Figure 5).

There was no relationship evident between the inflammatory proteins and the epimer 3epi25(OH)D in either cohort. 

Further, there was no relationship of 25(OH)D_3_ (Supplementary Figure S1) or 1,25(OH)2D3 (Supplementary Figure S2) with any of the MMPs.

## 5. Discussion

These data show that when controlled for obesity, in non-obese PCOS subjects that did not differ for either BMI or insulin resistance compared to controls, inflammatory proteins and matrix metalloproteinases were not elevated in PCOS in comparison with the matched controls. In addition, 25(OH)D_3,_ its epimer 3epi25(OH)D and the active 1,25(OH)_2_D_3_ did not correlate with any of the inflammatory proteins or the matrix metalloproteinases studied. Vitamin D deficiency can be exacerbated by obesity through decreased bioavailability, as vitamin D is sequestered in adipose tissue [38]; however, in this study the subjects were not obese, and the results clearly demonstrated that 25(OH)D_3,_ 3epi25(OH)D and the active 1,25(OH)2D3 did not correlate with BMI.

The presence of increased inflammation is clinically important and in PCOS it has been related to the underlying pathophysiological process of PCOS and its complications [6,9]. Whilst CRP has been reported to be elevated in both obese and non-obese PCOS [4,5,6], BMI was accounted for but insulin resistance was not; however, conversely, no difference in circulatory CRP between women with or without PCOS has been reported [7,8]. A recent systematic review with meta-analysis concluded that circulatory CRP is elevated in women with PCOS independent of obesity [9]; however, the contribution of insulin resistance was again not addressed, which is important as PCOS women may have elevated insulin and insulin resistance whilst being non-obese [44]. Other markers of inflammation including TNF alpha have been reported in non-obese women with PCOS, though again without accounting for insulin resistance [10]. It is recognised that the severity of PCOS may be aggravated with obesity, and it has been reported that women with PCOS present higher serum concentrations of TNF and C-reactive protein (CRP) as well as monocyte and lymphocyte circulating levels, with inflammatory infiltration in ovarian tissue [45]. This chronic inflammatory state is aggravated by obesity and hyperinsulinemia, and the combined impact of hyperinsulinemia, obesity, hyperandrogenism, and the inflammatory state has been reported [46]. Both obesity and hyperinsulinism promote the molecular mechanisms associated with higher androgen expression [47]. Data from many studies infer both correlative and causative association between higher activity of proinflammatory processes in adipose tissue and impaired insulin metabolism, insulin resistance, and type 2 diabetes [48]; thus, in obese individuals, there is a predominance of pro-inflammatory processes that causes systemic low-grade chronic inflammation that appears to not be present in non-obese and non-insulin resistant PCOS subjects. This suggests that, for further confirmation, this study needs to be repeated in a cohort of non-obese PCOS subjects that are insulin resistant and that are matched in BMI and age to a control population to determine the contribution of insulin resistance to chronic inflammation and whether this would then be modulated by vitamin D metabolites.

In obese women, there is an observed imbalance between classically activated macrophages (M1) and alternatively activated macrophages (M2). In these groups, there is a higher concentration of M1 macrophages. Macrophages represent the most abundant immune cell subtype in ovaries and adipose tissue, and they are essential for maintaining balance of destructive versus protective cell-mediated immunity that is an inherent component of the inflammatory process (14). Macrophage levels vary cyclically during the menstrual cycle, being greatest during ovulation and the luteal phase; this pattern implies that macrophages are under the hormonal regulation of progesterone (14). As noted above, PCOS women commonly present with obesity and insulin resistance, pathophysiological states characterised by macrophages transitioning from an anti-inflammatory M2 phenotype to a pro-inflammatory M1 phenotype (14). M1 macrophages are inflammatory because of the cytokines (TNF-a, IL-1, and IL-6 for example) that they produce; these cytokines are present in high concentration in both the serum and follicular fluid of PCOS women (14,15). The hyperandrogenism found in PCOS likely results in the conversion of macrophages to the M1 state, thereby increasing cytokine levels and amplifying PCOS symptoms such as insulin resistance, androgen production and the imbalance in the hypothalamic–pituitary–ovarian axis secretion (14,15). Whilst these PCOS women showed significant hyperandrogenaemia, it is evident that in the absence of differences in inflammatory parameters between the PCOS and control cohorts, that hyperandrogenaemia alone in the absence of obesity and insulin resistance did not contribute to chronic inflammation in its own right. This suggests that the association of hyperandrogenaemia to cytokine responses, and hence inflammation, may be as an epiphenomenon to the underlying insulin resistance in those studies (14,15).

In this cohort of non-obese, non-insulin resistant PCOS patients, inflammatory indices were not elevated in comparison to the control population, suggesting that the alterations seen for the inflammatory proteins and matrix metalloproteinases reported in PCOS are an epiphenomenon reflective of obesity and insulin resistance rather than inherent processes in PCOS. Both obesity and insulin resistance have been independently associated with inflammation [49] and indeed may be synergistic, particularly as insulin resistance is related to an increasing BMI [50]. It is recognised that many of the cardiovascular risk factors reported are linked to obesity rather than the underlying PCOS pathophysiology; however, determining the relative effects of insulin resistance, obesity and the inherent pathophysiology of PCOS [51,52] is complex. In addition, ethnicity may affect the underlying disease process of PCOS [53]. The contribution of obesity and insulin resistance were specifically addressed in this study to circumvent these confounders.

No correlation was seen for either 25(OH)D3, 3epi25(OH)D or the active 1,25(OH)2D3 levels with either the inflammatory proteins or matrix metalloproteinases. This data suggests that the changes in the inflammatory factors reported in PCOS are not contributed to or exacerbated by vitamin D levels, and therefore any deleterious effects that vitamin D deficiency may have in those with PCOS is not mediated through the inflammatory process. This is surprising as vitamin D deficiency has been associated with chronic inflammation [54] and changes in the complement system [55] that would enhance and be enhanced by inflammatory processes. Furthermore, vitamin D supplementation has been shown to reduce inflammation in PCOS women [56,57]. Conversely, it has been proposed that, as vitamin D deficiency can be seen in those with adequate sunlight, that it is chronic inflammation that may lead to a deficiency in vitamin D and all its sequelae that are reported in the literature [29]. How vitamin D may effect a protective mechanism in PCOS remains unclear, but its deficiency has been associated with hypertension and activation of the renin angiotensin system and low vitamin D levels have been linked to secondary elevation of PTH and elevated arterial resistance that leads to hypertension [58]. Additionally, vitamin D deficiency has been linked to elevated complement pathway proteins for both the classical and alternate cascades [59] together with complement activation. The development of hypertension with complement activation would initiate an inflammatory response that would be potentially exacerbated by the combination of obesity and insulin resistance, and what this study indicates is that those PCOS women who are non-obese and not insulin resistant may have the same cardiovascular risk as those without PCOS.

Overall, the major clinical importance of these findings is that the inflammatory state related to PCOS and its complications and the associated vitamin D deficiency are abrogated and avoided in those PCOS subjects that are non-obese and not insulin resistant, even in the presence of hyperandrogenaemia, thus likely preventing any PCOS related comorbidities. It remains to be seen whether, for those obese and insulin resistant subjects, all of their inflammatory related parameters reverse with substantial weight loss as reported for bariatric surgery, or whether a metabolic memory response remains and inflammation is unaltered.

In any study that describes negative findings such as this, a type 2 (or beta) statistical error due to inadequate sample size may be encountered. To address this, the power analysis was based on changes observed for TNF alpha in a non-obese PCOS population, and 24 rather than 12 subjects per group were recruited; however, as power analyses could not be specifically calculated for the other proteins, a type 2 (beta) error cannot be excluded, though there were no trends observed suggesting that such an error is unlikely. All the participants were Caucasian and therefore it is possible that the results presented here may not be generalisable to different ethnic groups, and indeed ethnic differences in levels of vitamin D and this epimer form may exist [34].

In conclusion, in a non-obese PCOS population matched for age, BMI and insulin resistance, circulating inflammatory proteins and matrix metalloproteinases were not elevated and did not correlate with 25(OH)D_3,_ its epimer 3epi25(OH)D or 1,25(OH)_2_D_3_ in either control or PCOS women, indicating that the inflammatory response is absent and the vitamin D-metabolite independent in non-obese non-insulin resistant PCOS.

## Figures and Tables

**Table 1:** Demographics, baseline, hormonal and metabolic parameters of the PCOS subjects and controls (mean ± SD).

	Control (n = 24)	PCOS (n = 24)
Age (years)	32.5 ± 4.1	31 ± 6.4
BMI (kg/m^2^)	24.8 ± 1.1	25.9 ± 1.8
Fasting glucose (mmol/L)	4.9 ± 0.4	4.7 ± 0.8
HbA1C (mmol/mol)	30.9 ± 6.5	31.8 ± 3.0
HOMA-IR	1.8 ± 1.0	1.9 ± 1.6
SHBG (nmol/L)	104.2 ± 80.3	71.7 ± 62.2
Free androgen index (FAI)	1.3 ± 0.5	4.1 ± 2.9 **
CRP (mg L^−1^)	2.34 ± 2.34	2.77 ± 2.57
AMH (ng/mL)	24 ± 13	57 ± 14 **
25 hydroxy vitamin D_3_ (nmol/l)	46.2± 23.5	54.0 ± 27.4
25-hydroxy-3epi-vitamin D_3_	0.97 ± 0.64	1.69 ± 1.36
1,25 dihydroxy vitamin D_3_ (ng/mL)	0.03 ± 0.02	0.04 ± 0.2

All parameters did not differ other than those marked ** = *p* < 0.01. BMI—body mass index; HbA1c—glycated haemoglobin; HOMA-IR—homeostasis model of assessment—insulin resistance; CRP—C reactive protein; SHBG—sex hormone binding globulin; AMH—anti-Müllerian hormone.

**Table 2:** Inflammatory protein levels in non-obese non-insulin resistant PCOS subjects and BMI-matched controls.

	Control	PCOS	*p* Value
IL5	247 (231)	252 (130)	0.92
AZU1	194 (120)	168 (52)	0.29
RPS6KA5	150 (39)	155 (52)	0.68
CD40LG	420 (480)	296 (128)	0.18
HMGB1	6397 (7451)	4024 (1283)	0.10
IL34	475 (86)	482 (86)	0.77
CXCL10	1546 (392)	1632 (438)	0.44
S100A9	8943 (4519)	7103 (2911)	0.07
CCL19	424 (597)	365 (114)	0.61
SIGLEC1	91 (39)	101 (33)	0.33
TBK1	170 (59)	166 (51)	0.77
CLEC7A	683 (413)	654 (298)	0.76
PRKCZ	1079 (1513)	742 (627)	0.27
IL1B	2492 (1057)	2316 (785)	0.48
IL10RB	1414 (2157)	924 (188)	0.23
PTGS2	885 (674)	760 (273)	0.36
FGF8	621 (357)	584 (140)	0.60
IL1A	636 (280)	584 (173)	0.40
IL6	269 (69)	298 (127)	0.30
IL10	290 (589)	146 (26)	0.19
IL12	198 (53)	213 (105)	0.49
IFN-g	552 (156)	804 (906)	0.15
TNF-a	344 (89)	352 (58)	0.67
IL17	412 (91)	462 (205)	0.23
MMP9	32,886 (19,126)	29,041 (15,122)	0.40
MMP3	428 (117)	720 (1264)	0.22
MMP7	1027 (299)	939 (284)	0.25
MMP17	959 (817)	698 (203)	0.10
MMP2	6508 (1628)	6492 (1399)	0.97
MMP12	883 (405)	833 (312)	0.60
MMP1	975 (756)	859 (452)	0.48
MMP13	684 (696)	527 (184)	0.25
MMP14	1404 (1802)	1007 (373)	0.25
MMP16	671 (301)	575 (73)	0.10
MMP10	750 (325)	823 (336)	0.41
MMP8	3439 (2877)	2500 (1175)	0.11

Data are presented as mean ± 1 standard deviation of relative fluorescent units (RFU). C-X-C motif chemokine 10 (CXCL10), Interleukin-5 (IL5), Azurocidin (AZU1), C-type lectin domain family 7 member A (CLEC7A), Serine/threonine-protein kinase (TBK1), Protein kinase C zeta type (PRKCZ), Ribosomal protein S6 kinase alpha-5 (RPS6KA5), CD40 ligand (CD40LG), Interleukin-34 (IL34), High mobility group protein B1 (HMGB1), Protein S100-A9 (S100A9), Interleukin-1 beta (IL1B), C-C motif chemokine 19 (CCL19), Sialoadhesin (SIGLEC1) and Interleukin 10 receptor beta subunit (IL10RB). Prostaglandin G/H synthase 2 (PTGS2), Fibroblast growth factor 8 (FGF8), Interleukin (IL), Interferon gamma (IFN-g), Tumour necrosis factor alpha (TNF-a), Matrix metalloproteinase (MMP).

## Data Availability

Data presented in this study are available on request from the corresponding author. The data are not publicly available due to privacy.

## Supplementary Materials

The following supporting information can be downloaded at: https://www.mdpi.com/article/10.3390/nu14173540/s1, Figure S1: No relationship was found between Vitamin D_3_ and the matrix metalloproteinases (MMPs) MMP1, MMP2, MMP3, MMP7, MMP8, MMP9, MMP10, MMP12, MMP13, MMP14, MMP16 and MMP17, Figure S2: No relationship was found between 1,25(OH)_2_D_3_ and the matrix metalloproteinases (MMPs) MMP1, MMP2, MMP3, MMP7, MMP8, MMP9, MMP10, MMP12, MMP13, MMP14, MMP16 and MMP17.
