# Supplementary material for: Inflammatory Markers in Non-Obese Women with Polycystic Ovary Syndrome Are Not Elevated and Show No Correlation with Vitamin D Metabolites
Source: Nutrients. 2022 Aug 27;14(17):3540. doi: 10.3390/nu14173540 (PMC9459875; doi:10.3390/nu14173540)

Supplementary Figure S1

25(OH)D3, no correlation with MMPs

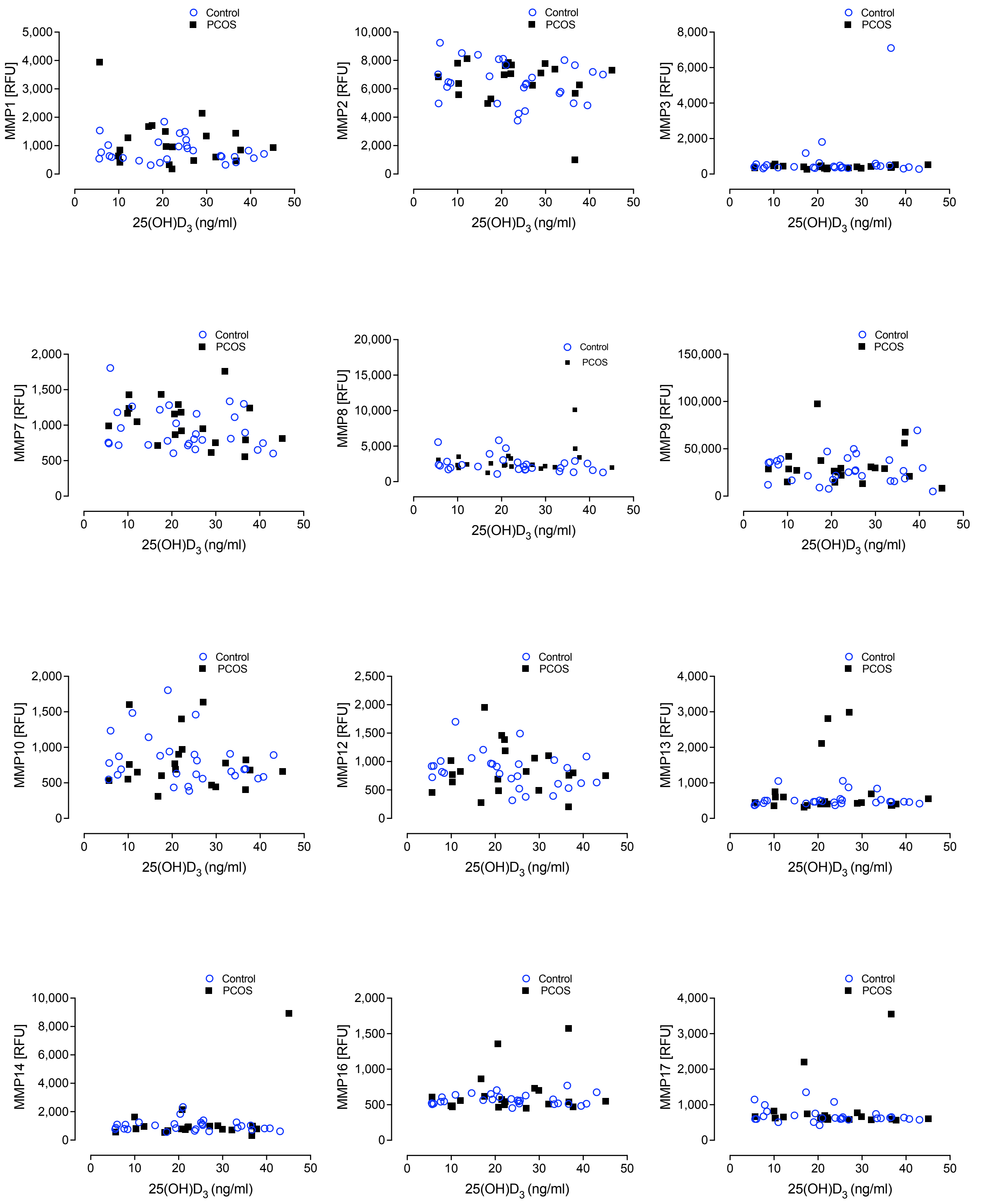

Supplementary Figure S2

1,25(OH)2D3, no correlation with MMPs

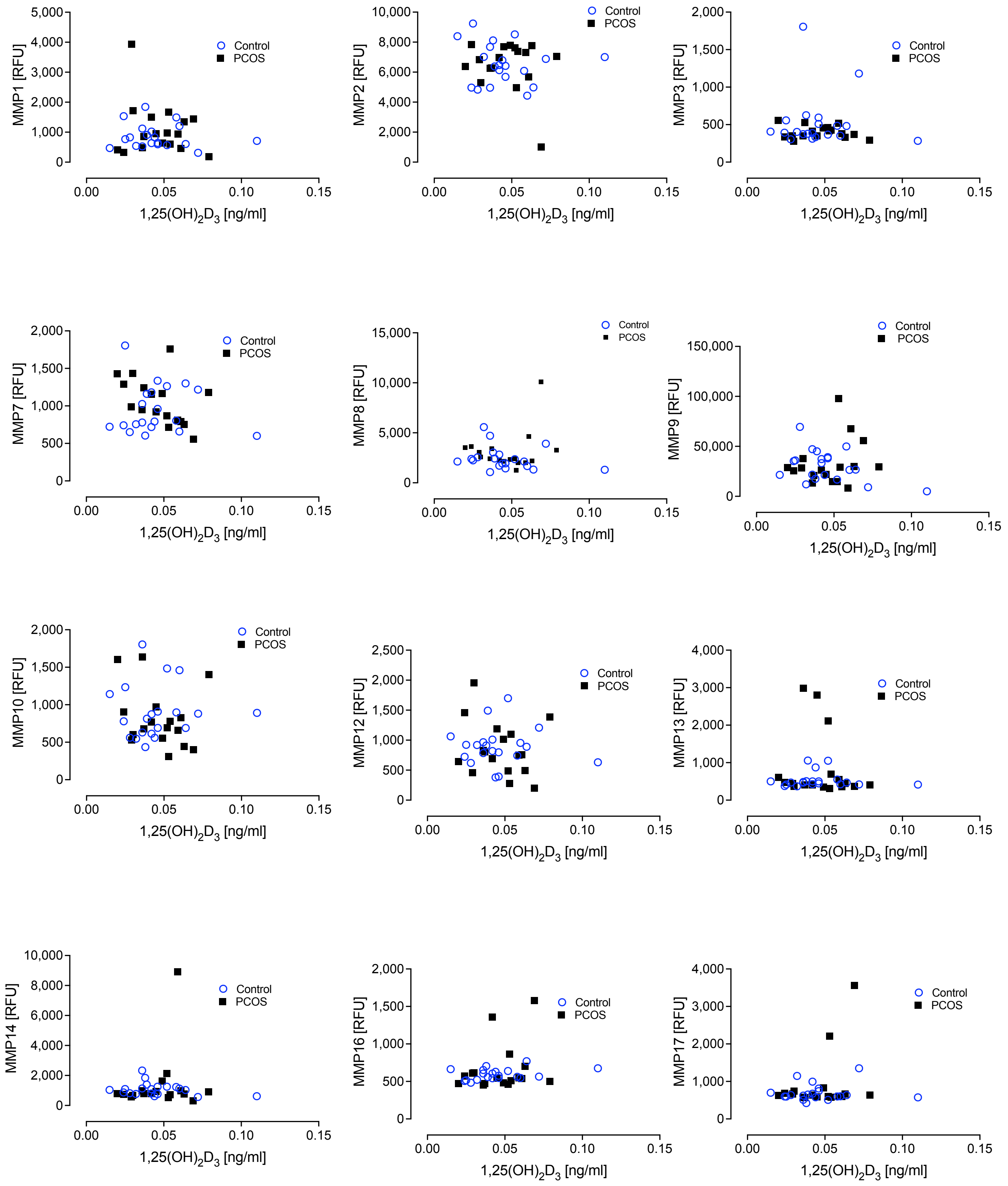

Supplement: Supplementary file 1 [file nutrients-14-03540-s001.zip › nutrients-1885560-supplementary.pdf]
